# *Enterococcus* contamination of infant foods and implications for exposure to foodborne pathogens in peri-urban neighbourhoods of Kisumu, Kenya

**DOI:** 10.1017/S0950268824000062

**Published:** 2024-01-24

**Authors:** Fanta D. Gutema, Oliver Cumming, Jane Mumma, Sheillah Simiyu, Edwin Attitwa, Bonphace Okoth, John Denge, Daniel Sewell, Kelly K. Baker

**Affiliations:** 1 Department of Occupational and Environmental Health, University of Iowa, Iowa City, IA, USA; 2 Department of Microbiology, Immunology and Veterinary Public health, Addis Ababa University, Bishoftu, Ethiopia; 3 Department of Disease Control, London School of Hygiene and Tropical Medicine, London, UK; 4 Center of Research, Great Lakes University of Kisumu, Kisumu, Kenya; 5 African Population and Health Research Center, Nairobi, Kenya; 6 Department of Biostatistics, University of Iowa, Iowa City, IA, USA

**Keywords:** *enterococcus*, foodborne pathogens, food hygiene, food safety, infant foods

## Abstract

We collected infant food samples from 714 households in Kisumu, Kenya, and estimated the prevalence and concentration of *Enterococcus*, an indicator of food hygiene conditions. In a subset of 212 households, we quantified the change in concentration in stored food between a morning and afternoon feeding time. In addition, household socioeconomic characteristics and hygiene practices of the caregivers were documented. The prevalence of *Enterococcus* in infant foods was 50% (95% confidence interval: 46.1 - 53.4), and the mean log_10_ colony-forming units (CFUs) was 1.1 (SD + 1.4). No risk factors were significantly associated with the prevalence and concentration of *Enterococcus* in infant foods. The mean log_10_ CFU of *Enterococcus* concentration was 0.47 in the morning and 0.73 in the afternoon foods with a 0.64 log_10_ mean increase in matched samples during storage. Although no factors were statistically associated with the prevalence and the concentration of *Enterococcus* in infant foods, household flooring type was significantly associated with an increase in concentration during storage, with finished floors leading to 1.5 times higher odds of concentration increase compared to unfinished floors. Our study revealed high prevalence but low concentration of *Enterococcus* in infant food in low-income Kisumu households, although concentrations increased during storage implying potential increases in risk of exposure to foodborne pathogens over a day. Further studies aiming at investigating contamination of infant foods with pathogenic organisms and identifying effective mitigation measures are required to ensure infant food safety.

## Introduction

Infants need extra energy and nutrients complementary to breastfeeding for optimal growth and development. To meet these nutritional demands, nutritionists recommend providing infants with diversified diets beginning at six months of age [1]. Infants, especially those between 6 and 12 months, are vulnerable to diarrheal illnesses when exposed to contaminated foods [2]. Globally, foodborne diseases are a major public health problem, with children living in low-income countries bearing the highest burden [2, 3]. The World Health Organization estimates that children under 5 years old bear 40% of the foodborne disease burden with 125,000 deaths occurring annually. Diarrheal diseases account for the largest share of the foodborne attributable disease burden with the highest burden in Africa [2]. Several factors contribute to the high foodborne disease burden, including lack of access to safe water; sanitation, and hygiene [4–6] in food production, packaging, distribution, and preparation settings [7, 8]; lack of refrigeration for food storage [9]; informal marketing of foods [10]; and lack of food safety regulation systems that contribute to contamination and re-contamination of foods [11].

Infants can be exposed to foods that are contaminated with pathogens in ready-to-eat packaged or purchased foods as well as pathogens introduced during preparation and storage processes at home [12, 13]. Several studies have demonstrated the risk of exposure to foodborne pathogens through consumption of common supplemental infant foods, like cow milk, soy protein-based formulas, cereals, and pureed fruits and vegetables [14–17].

However, there is a paucity of information on the microbial quality and safety of the different types of products sold as infant foods and homemade infant foods in resource-limited countries. Assessment of the microbial contamination of infant foods is required to design appropriate food system intervention measures [18]. Factors ascribed to household contamination of infant foods by caregivers include, but are not limited to, unhygienic food handling practices during preparation [19], lack of handwashing practices at critical times [20–22], use of contaminated water for food preparation [23], and lack of access to an improved latrine [24, 25]. Unsafe storage of infant foods for several hours at a temperature conducive for microbial growth and multiplication, without adequate heat treatment, can increase microbial concentrations to unsafe levels for feeding a child [26, 27].

In Kenya, diarrhoea is a common cause of death with a mortality rate of 122 per 100,000 in children less than 5 years [28] and a high (26.6%) prevalence in infants between 6 and 12 months [29]. It has been estimated that half of these diarrheal cases are due to foodborne bacterial infections [30]. Our prior research showed that the type of infant food was significantly associated with the presence and diversity of enteric pathogens in infant weaning foods [17, 31]. However, infant food is frequently made in the morning by caregivers and stored over the day at ambient temperatures for re-feeding, suggesting exposure risks from food can change over time and may be heavily influenced by storage conditions. Prior studies did not track food over time to untangle how morning food preparation conditions versus storage conditions contribute to microbial presence and increase in concentration. Given its high survival rate in the environment and capacity to withstand harsher food treatment conditions (e.g., heating), *Enterococcus* indicator organism [32] provides unique information about food contamination compared to the commonly used *Enterobacteriaceae*, total aerobic bacteria, and coliform indicator organisms [23, 33–35]. Therefore, the objectives of this study were to (1) estimate the prevalence and concentration of *Enterococcus* bacteria, an indicator of hygienic conditions, in different types of infant foods in Kisumu, Kenya, (2) assess risk factors for contamination presence and concentration during morning food preparation and feeding, and (3) assess risk factors for increased concentration of *Enterococcus* during short-term storage of infant foods for repeat feeding. Knowledge of the risk factors that contribute most to microbial contamination of infant foods is important for designing tailored interventions to reduce the burden of foodborne diseases in infants.

## Materials and methods

### Study settings and design

The study was conducted from 2018 to 2019 in low-income peri-urban neighbourhoods of Nyalenda A and Nyalenda B in Kisumu, Kenya. The study was part of the Safe Start cluster randomized controlled trial of an infant food hygiene behaviour change intervention in Kisumu, Kenya (Clinical Trials identifier: NCT03468114). Kisumu is a city situated in the western region of Kenya and has an estimated population of 1,224,531 according to Kisumu County integrated development plan 2018–2022 [36]. The detailed eligibility inclusion and exclusion criteria for enrolling caregivers and other information about the study setting and study design were described in previous studies [17, 37–40]. Briefly, infants aged 22 weeks (+/− 1 week), verified by reviewing the infant’s birth identity card, who permanently resided in the study neighbourhoods, and their primary or secondary caregiver aged 18 years of age or older, were eligible for enrolment. Caregivers with medical, psychological, or social conditions that would impede their ability to provide informed consent were excluded. Enrolled caregivers and their infants participated in the study for 3 months. During the clinical trial, food hygiene data were collected at 6 (baseline), 8 (midline), and 9 (end line) months of infant’s age.

The sample size available for this study was based upon calculations needed for the Safe Start cluster randomized controlled trial. Of the 898 infant and caregiver who were enrolled, we used baseline household survey responses and microbial data from infant food samples from 714 households who completed the 8-month infant age midline follow-up visit. In a subset of 212 households, food samples were collected at two occasions: in the morning and in the afternoon. A structured questionnaire was used to collect data on (1) socioeconomic characteristics of the caregivers, (2) types of infant foods and storage conditions, and (3) self-reported handwashing practices at various critical points.

### Ethical statement

Community health volunteers of the participating communities facilitated the enrolment process, and trained enumerators collected survey data and samples after obtaining written informed consent from the child’s primary caregiver for participation in the study. A copy of the informed consent was provided to the participant for their permanent records. Original study records are stored on a secured server as a file requiring password access, which is only available to a few members of the study leadership team. A de-identified dataset generated for analytical purposes was used for this analysis. The study was approved by the scientific and ethical review committees at Great Lakes University of Kisumu (GLUK) (Ref. No. GREC/010/248/2016), London School of Hygiene and Tropical Medicine (LSHTM) (Ref. No. 14695), and University of Iowa (IRB ID 201804204).

### Study variables

The study outcomes are (1) prevalence of *Enterococcus* in all 714 infant foods in Kisumu, (2) concentration of *Enterococcus* in all 714 infant foods, (3) the change in concentration of the bacteria between food prepared in the morning for first feeding and the remaining food in the afternoon after storage for the 212 subgroup of households. Potential factors of interest included caregivers’ marital status, education level, household wealth quintile, number of infants in the household, infant food types, food storage condition, household flooring, water source, sharing a house with animals, handwashing area with soap and water, and handwashing practices at various critical times. The critical times of handwashing practices include handwashing after self-defecation, cleaning defecated infant, and contacting animals, before feeding infants, eating, and preparing foods.

### Sample collection and processing

Foods, including liquids, intended for infant feeding at the time of visits were collected by requesting the caregivers to transfer a small portion (~5 grams or millilitres) into a sterile labelled 250 ml Whirl Pak (Sigma-Aldrich, St. Louis, MO, USA). These were transported to the GLUK laboratory using an icebox containing ice packs and processed within 24 h of collection to detect and enumerate *Enterococcus* spp., following the procedures described in the protocol paper [39]. Briefly, 1 ml, 0.1 ml, and 0.01 ml of original sample were homogenized in 30 mL of sterile phosphate-buffered saline (PBS) and vacuum filtered through a 0.45 μm pore size membrane filter (Millipore Corp., Bedford, MA, USA). The filters were cultured overnight on a selective medium, Slanetz and Bartley *Enterococcus* Medium (OXOID CM0377). For solid foods, five grams of original samples were first homogenized with 45 ml PBS. Then, 1 ml, 0.1 ml, and 0.01 ml dilutions were filtered and cultured on the *Enterococcus* agar plates. All the plates were incubated at 41.5 °C for 24 h [41]. After incubation, all light and dark red colonies were counted as *Enterococcus* and expressed as colony-forming units (CFU) present per gram or ml of food sample. A 30 ml volume of PBS used to resuspend food samples and wash membrane filters was processed each day as a negative control. Our approach to estimating concentration for a sample was to use the highest volume filtered with countable colonies (between 20 and 250 colonies per plate) to estimate concentration per mL/gram. If countable plates were from 0.1 or 0.01 mL volumes, and the higher volume in the series was too numerous to count (TNTC), then colony counts were multiplied by 10- or 100-fold dilution factor, respectively, to achieve a count per mL denominator. Any samples with inconsistent colony count across the sample volume series were determined positive, but concentration inconclusive.

### Data analyses

Data entry and data cleaning were performed using a Microsoft Excel spreadsheet and all the analyses were performed using R software version 4.1.2 (R Foundation for Statistical Computing, Vienna, Austria). Prior to analysis, the data collected during the trial were assessed for completeness and consistency by calculating the frequencies of each variable. Only those observations with complete information for each respective variable were included in the analysis. Descriptive statistics such as frequency and percentage were used to summarize the data, and a frequency table was used to present the results. Pearson chi-square test was used to assess the possible association between socioeconomic characteristic variables of the caregivers and infant food type.

Logistic regression analysis was used to assess the association between the prevalence of *Enterococcus* in all 714 food samples and the potential risk factors. Due to the observed low frequencies, fruit, potato, and tea food types were grouped together for better convergence of regression analyses. Similarly, infant food types containing milk such as only milk, porridge containing milk, and tea containing milk were grouped as ‘milk and milk-based foods’.

Candidate variable selection was made by running backwards, forwards, and stepwise selection models. The Akaike information criterion (AIC) score of each selection process was noted and the model with the lowest AIC score was selected as the best fit model [42]. The CFU of *Enterococcus* in foods were first transformed into log_10_, and the mean log_10_ of the CFU was computed to estimate the concentration of *Enterococcus* in infant foods sampled in the morning and afternoon. The change in the concentration between the same infant food collected at both afternoon and morning times was calculated by subtracting the morning concentration from afternoon concentration in the matched sample pair. Variation in the mean log_10_ *Enterococcus* concentration among the different types of infant foods was assessed using one-way analysis of variance. Due to significant left censoring of the *Enterococcus* distribution, Tobit regression analysis was used to assess the association of *Enterococcus* concentration in all infant foods with potential risk factors [43]. In samples that yielded no *Enterococcus* bacteria, the log_10_ of the concentration was considered as left-censored as they fell below the 1 CFU/mL/g detection limit of the method [44].

Among the households where the same food source was available for sampling at both the morning point of preparation and an afternoon feeding, the frequency of foods with decreased *Enterococcus* concentration during storage was low. Therefore, the change in the *Enterococcus* concentration was modelled using logistic regression with binary outcomes of increased concentration during storage versus the combined group of no change or decreased concentration during storage. A *p* value of less than 0.05 was set as a significance level for all analyses.

## Results

### Socioeconomic characteristics and handwashing practices

Among the caregivers, the majority were married (89.1%), had no refrigerator (85.2%), and had more than one young child (62.9%) (Table 1). More caregivers attended higher level education (46.1%) than any other level. In 70% of the cases, their house floor was covered with carpet or vinyl. Of the 714 households, infant foods were collected during morning food preparation and at later afternoon feedings in 29.7% (n = 212) of the households, while 6.2% (n = 44) had morning samples only and 64.1% (n = 458) had afternoon samples only. Foods that were prepared in the morning were fed to the infants in the morning, and any leftovers were stored and then fed in the afternoon. We identified nine different food types being fed to 8-month infants that included porridge, porridge containing milk, fruit, potato, milk, milk containing tea, tea, and cooked and uncooked grains (cereal). Porridge was the dominant (61.2%) food type followed by milk and milk-based foods (31.8%). Most of the caregivers did not report handwashing after cleaning defecated infant (59.5%), after handling animals (97.5%), and before preparing foods (61.2%), while 90.6% of them reported washing their hands before feeding infants. Prior to model development, we evaluated whether wealth was likely to be an important confounder of associations between different household hygiene factors and food contamination. The type of infant foods did not significantly vary based on the household wealth index (Pearson chi-square = 9.06, *p* = 0.697). However, increased wealth was associated with having a refrigerator (Pearson chi-square = 293.36, *p* < 0.001), the household having finished floors (Pearson chi-square = 70.302, *p* < 0.001), and higher maternal education level (Pearson chi-square = 129.67, *p* < 0.001). Household wealth index was also associated with infant caregivers’ handwashing practice after self-defecation (Pearson chi-square =17.12, *p* = 0.002) and before eating (Pearson chi-square =10.6, *p* = 0.032). However, in the 212 households where food samples were collected twice a day, none of the handwashing practices were not associated with the wealth index of the households (*p* > 0.05). Therefore, wealth was included as a confounder in subsequent analysis.

**Table 1.** Socioeconomic characteristics and handwashing practices of infant caregivers (N = 714) in peri-urban settlements of Kisumu, Kenya

Variables		Frequency	Percentage
Socioeconomic characteristics			
Marital status	Married	636	89.1
	Single	78	10.9
Have multiple children	No	265	37.1
	Yes	449	62.9
Education level	Primary or no education	274	38.4
	Secondary	111	15.5
	Higher	329	46.1
Household wealth index	Poorest	135	18.9
	Low middle	131	18.4
	Middle	152	21.3
	Upper middle	152	21.3
	Highest quintile	144	20.2
Food type	Fruit, potato, and tea	24	3.4
	Grains	26	3.6
	Milk and milk-based foods	227	31.8
	Porridge	437	61.2
Own refrigerator	No	608	85.2
	Yes	106	14.8
Household flooring type	Unfinished floor with visible dirt	56	7.8
	Unfinished floor covered with carpet or vinyl	498	69.7
	Finished floor with ceramic, concrete or tiles	160	22.4
Primary water source	Limited or unimproved	7	1
	Basic drinking water source	707	99
Sharing a house with animals	No	633	88.7
	Yes	81	11.3
Handwashing area with soap and water	No	355	49.7
	Yes	359	50.3
Latrine type	Unimproved latrine	93	13
	Improved latrine	621	87
Self-reported handwashing practice			
After cleaning an infant that defecated	No	425	59.5
	Yes	289	40.5
After self-defecation	No	98	13.7
	Yes	616	86.3
After handling animals	No	696	97.5
	Yes	18	2.5
Before feeding a child	No	67	9.4
	Yes	647	90.6
Before eating	No	292	40.9
	Yes	422	59.1
Before preparing food	No	437	61.2
	Yes	278	38.9

### Prevalence and concentration of enterococcus in infant foods

The prevalence of *Enterococcus* in infant foods across the 714 participating households was 50% (95% confidence interval: 46.1-53.4), with a mean log_10_ CFU of 1.1 (SD +1.4). The prevalence was highest in grains (65.4%) followed by milk and milk-based foods (52%), the group of fruit, potato, and non-milk teas (50%), and porridge (48%). However, the difference in the prevalence of *Enterococcus* was not statistically significant among the food types (*p* > 0.05). Handwashing before feeding a child and handwashing after handling animals were retained in the final logistic regression model. However, neither variable was significantly associated (*p* < 0.05) with the prevalence of *Enterococcus* in infant foods. The mean log_10_ *Enterococcu*s concentration was highest in cooked and uncooked grains followed by porridge, milk and milk-based foods, and combination of fruit, potato, and tea (Table 2). However, the variation was not statistically significant (*p* = 0.501). Similar to prevalence results, none of the variables were associated with *Enterococcus* concentration in infant foods (Table 3).

**Table 2.** The concentration of *Enterococcus* (mean log_10_ CFU/ml/g) in infant foods in peri-urban neighbourhoods of Kisumu, Kenya

Food types	Number (%) of positive	Mean
Fruit, potato, and tea	13 (54.2)	0.94
Grains	17 (65.4)	1.23
Milk and milk-based foods	123 (54.2)	1.07
Porridge	229 (52.4)	1.18

**Table 3.** Tobit regression model for association of the potential risk factors with *Enterococcus* concentration (log_10_ CFU/ml/g) in infant foods in peri-urban neighbourhoods of Kisumu, Kenya

Variables	Coefficient (95%CI)	*p* value
Socioeconomic characteristics		
Own refrigerator (yes vs no)	0.1 (−0.4, 0.6)	0.8
Marital status (single vs married)	0.1 (−0.5, 0.7)	0.69
Have multiple children (yes vs no)		0.43
Education level		
Secondary vs primary or no education	0.3 (−0.2, 0.9)	0.27
Higher vs primary or no education	0.2 (−0.2, 0.6)	0.36
Food type		
Grains vs fruit, potato, and tea	0.6 (−0.8, 1.9)	0.44
Milk and milk-based foods vs fruit, potato, and tea	0.3 (−0.8, 1.3)	0.6
Porridge vs fruit, potato, and tea	0.1 (−0.9, 1.1)	0.84
Household flooring type		
Unfinished covered floor vs unfinished dirt floor	0.3(−0.4, 1.1)	0.39
Finished floor vs unfinished dirt floor	0.6(−0.1, 1.3)	0.11
Primary water source		
Basic drinking water source vs limited or unimproved source	−0.4 (−2.3, 1.5)	0.69
Sharing a house with animals (yes vs no)		0.6
Household wealth index		
Low-middle vs poorest	−0.2 (−0.8, 0.4)	0.44
Middle vs poorest	−0.6 (−0.8, 0.4)	0.48
Upper-middle vs poorest	−0.01 (−0.6, 0.6)	0.98
Highest quintile vs poorest	0.1 (−0.5, 0.6)	0.79
Handwashing area with soap and water (yes vs no)	−0.1 (−0.5, 0.2)	0.51
Latrine type		
Unimproved latrine vs improved latrine	−0.02 (−0.6, 0.5)	0.94
Handwashing practice		
After cleaning an infant that defecated (yes vs no)	0.1 (−0.3, 0.48)	0.57
After self-defecation (yes vs no)	0.2 (−0.3, 0.7)	0.48
After handling animals (yes vs no)	0.4 (−0.7, 1.5)	0.49
Before feeding (yes vs no)	0.4 (−0.2, 1.1)	0.2
Before eating (yes vs no)	0.2 (−0.1, 0.6)	0.16
After preparing food (yes vs no)	0.1 (−0.3, 0.4)	0.8

Abbreviations: CI, confidence interval.

### Storage effect on concentration of enterococcus

In the 212 households where food samples were collected twice a day, the prevalence of *Enterococcus* contamination in food samples collected in the morning (32%) was lower than in the afternoon (40%). *Enterococcus* detection or non-detection was concordant in morning and afternoon samples for 83.5% (177/212) of food samples. In 12.3% (26/212), detection occurred only in the afternoon after being stored during the day, while only 4.2% (9/212) of the samples were contaminated in the morning pre-storage but not in the afternoon. The overall mean log_10_ CFU of *Enterococcus* concentration was 0.47 in the morning and 0.73 in the afternoon foods, and between matched pairs, there was a mean increase in concentration of 0.64 log_10_ (range: 0–5) during storage. Among the variables considered in this study, only household with finished flooring type was significantly associated with an increase in *Enterococcus* concentration in infant foods during storage (Table 4).

**Table 4.** Factors associated with increase in *Enterococcus* concentration during storage in infant foods collected from 212 households in Kisumu, Kenya

Variables	Number sampled	N (%) foods with increased *Enterococcu*s concentration	Adjusted OR (95% CI)	*p* value
Household flooring type Unfinished dirt floor	25	4 (16.0)	Ref^a^	
Unfinished covered floor	40	8 (20.0)	1.6 (0.4, 7.1)	0.51
Finished floor	147	54 (36.7)	3.8 (1.3, 15)	0.03
Handwashing after handling animals No	206	63 (30.6)	0.3 (0.0, 1.6)	0.13
Yes	6	3 (50.0)	Ref	

Abbreviations: CI, confidence interval; OR, odds ratio.

a Reference category.

## Discussion

Our study demonstrated that *Enterococcus* presence in infant foods in low-income households in Kisumu was common, although its presence could not be attributed to any specific infant food and household characteristics. Additionally, contamination levels increased when food was stored for repeat feeding during a day. Counterintuitively, we found that finished floors were associated with an increase in microbial concentration during storage. Our results confirm that many infants in these neighbourhoods of Kisumu ingest food containing faecal indicators, implying a potential risk of exposure of infants to foodborne diseases needing intervention.

Reports on the prevalence and concentration of *Enterococcus* in infant foods are limited. However, the 50% prevalence of *Enterococcus* in infant foods observed herein was comparable with the 53% (N = 58) prevalence reported among children in an urban setting in Maputo, Mozambique [18]. Although we observed no significant variation based on food types, the prevalence of *Enterococcus* contamination was considerable in all food types with a slightly higher detection frequency in grains. Our prior work in Kisumu also found a similar prevalence of *Enterobacteriaceae* detection across food types at the point of infant feeding, regardless of microbial safety of food products used to make the food [12, 17, 31]. Both microbial indicators indicate that multiple types of infant foods are handled under unhygienic conditions in Kisumu.

The observed 1.1 mean log_10_ *Enterococcus* concentration was low compared to the other microbiological criteria set for infant foods. The concentration was low when compared with the maximum limit of aerobic plate count established by the CODEX (4 log CFU/g) for dried and instant infant products [45] and the mean *Enterococci* count of 854 CFU/g in infant foods [18]. In contrast, it was high compared to the standard microbial limit of 0 per 100 ml of drinking water for *Enterococcus* set by several countries to determine water quality [46]. The Codex Alimentarius Commission set no detection limit in 10 g of infant formula for general *Enterobacteriaceae* [47]. However, there are no specific guidelines elsewhere on the standard limit for *Enterococcus* to regulate the hygienic quality and safety of infant foods. As result, it is not possible to conclude whether the observed concentration exceeds safety limits. Left-censored distributions with low levels of contaminates is common in environmental studies, including in our prior studies of *Enterobacteriaceae* and enteric pathogens in Kisumu [12, 17, 31]. Further studies are required to determine the maximum detection limit of microbial safety indicators in infant foods, their correlation with pathogenic organisms, and the risk of consumption of *Enterococcus* contaminated foods to include in international and national infant food safety guidelines.

This study expands upon our prior studies by examining temporal changes in contamination concentration and the conditions that explain those changes. In the present study, we observed a significant increase in the concentration of *Enterococcus* particularly in households with finished floor type with ceramic, concrete, or tiles during storage. Finished floors was almost perfectly predicted by higher wealth level, and the fact that 69.4% of our 212 households with food available for afternoon collection had finished floors vs 22.4% of the overall study population indicates that our ability to sample stored food was biased by those household possessing greater wealth. Nonetheless, one explanation for these results is that caregivers may perceive that finished floors are safer and cleaner than others and therefore be more negligent about protecting food from contamination. Alternatively, high levels of contamination may be inevitable in even the wealthiest household due to poor handwashing or other conditions, with food contamination levels potentially being even higher in sampled foods in more households with unfinished floors. However, as observed in this study, all the handwashing practices were not associated with the wealth index of the households, suggesting the need for further investigation of other conditions that have contributed to contamination of infant foods in households with finished floor. The increase in the concentration of *Enterococcus* could also be attributed to the overall effect of unhygienic handling practices after food preparation and poor storage conditions. Better adherence to preparation practices, such as cleaning food preparation areas, thorough washing/rinsing of grains, fruits, and vegetables, and adequate heating and reheating of foods can prevent infants from ingesting contamination derived from the food production, packaging, and distribution supply chain, as well as eliminate contamination from the household food preparation environment [1].

While our 212 households were wealthier than many of their neighbours, the majority (85.2%) had no refrigerator for cold storage of those foods, which could explain the increase in concentration after morning preparation. Storage of foods at room temperature favours the growth and multiplication of microorganisms. In the absence of a refrigerator, immediate feeding of freshly cooked infant foods within 2 h or re-heating stored foods to the 70 ^o^C level that adequately kills pathogenic organisms is required to ensure food safety, especially in unhygienic households [48]. Although we observed an increase in contamination during storage in most of the food samples tested, in few cases (9/212), *Enterococcus* was not re-detected in foods sampled in the afternoon that were positive earlier in the morning. The absence of bacteria in these households could be due to a caregiver refrigerating or reheating food to a bactericidal temperature.

The lack of handwashing after handling animals (97.5%) and before preparing foods (61.2%) could lead to transmission of pathogens from animals to humans via contamination of food during preparation or storage [49, 50]. Interestingly, most of the caregivers reported that they wash their hands after toilet visits and before feeding children. These are important practices that need to be promoted to minimize the role of caregivers as a source of pathogens and direct contamination of infant foods, as well as handwashing at other critical times.

Overall, this study has three major public health implications. First, the occurrence of *Enterococcus* in infant foods suggests the potential for contamination of infant foods with pathogenic organisms. *Enterococcus* is an indicator of contamination by either human or animal faecal materials [32] and can be transmitted through food alongside foodborne pathogens [51]. Previous studies indicated a strong association of contact with animal and/or human faces and the occurrence of various pathogenic organisms in infant foods [17, 52, 53].

Contamination sources could be derived from food production animals, household animals, and human faces contamination on household surfaces and hands, and thus, it is essential to assess the entire point to identify the most likely sources for microbial presence and increases in concentration. Future studies should focus on identifying sources of food contamination with enteropathogens for tailored intervention.

Second, our results raise concerns about whether *Enterococcus* recovered from infant foods could harbour antimicrobial resistance genes and contribute to the spread of antimicrobial resistance genes in the study areas. *Enterococcus* are known for their natural inherent resistance to several antimicrobials and rapidly acquiring virulence and multidrug resistance determinants [54–57]. Third, *Enterococcus* in infant foods may pose a risk of illness to infants. *Enterococcus* spp have been traditionally considered non-pathogenic organisms, but some studies have reported that *Enterococcus* can cause food poisoning [56, 58, 59]. Their pathogenic potential needs further critical evaluation.

Our study has three limitations. First, this study depends on the caregiver’s self-report of handwashing practices, which might have resulted in information bias. Direct observation may have provided more valid assessments of handwashing practices among caregivers and associations with microbial contamination of infant foods. The lack of association between household hygiene factors and presence of contamination may reflect the importance of other conditions that were unmeasured during data collection, such as cleanliness of cooking and eating surfaces, dishes, and utensils. Second, our analysis of contamination levels during storage was limited by only one-thirds of households having stored infant food for short term for afternoon feeding and resulted in analysis skewed toward the wealthier subset of this population. Additionally, the high frequency of contamination-negative and low contamination samples resulted in zero-inflated distributions, leaving fewer matched morning and afternoon sample pairs to serve as contamination-positive outcomes for analysis. Third, our study included only participants from two sites of low-income peri-urban neighbourhoods. The conclusion from this study might not represent the actual conditions of the rural households and middle- and high-income urban neighbourhoods in Kisumu.

In conclusion, our study showed that infants were fed with various food types contaminated with *Enterococcus* at varying levels of prevalence and concentration, implying potential exposure of infants to foodborne pathogens and antimicrobial-resistant *Enterococcus.* Although most of the risk factors considered in this study were not significantly associated with *Enterococcus* contamination, some of the socioeconomic factors and handwashing practices of caregivers are concerning and indicate a need for public education promoting handwashing practices at all critical points and access to refrigeration for storage of prepared foods. Further studies aiming at identifying the sources and the risk factors and and quantifying the risk of contamination of infant foods with common foodborne pathogenic organisms and exploring effective and feasible risk mitigation measures are needed to ensure infant food safety.

## Data Availability

The authors confirm that the data needed to replicate the findings of this study are available within the manuscript.
